# GLA:D® Back Australia: a mixed methods feasibility study for implementation

**DOI:** 10.1186/s12998-022-00427-3

**Published:** 2022-04-07

**Authors:** Matthew Fernandez, Anika Young, Alice Kongsted, Jan Hartvigsen, Christian Barton, Jason Wallis, Peter Kent, Greg Kawchuk, Hazel Jenkins, Mark Hancock, Simon D. French

**Affiliations:** 1 School of Health, Medical and Applied Sciences, Central Queensland University, Brisbane, Australia; 2 Department of Chiropractic, Faculty of Medicine, Health and Human Sciences, Macquarie University, Sydney, Australia; 3 Department of Sports Science and Clinical Biomechanics, University of Southern Denmark, Odense, Denmark; 4 Chiropractic Knowledge Hub, Odense, Denmark; 5 La Trobe Sport and Exercise Medicine Research Centre, School of Allied Health, Human Services and Sport, La Trobe University, Melbourne, Australia; 6 Department of Surgery, St Vincent’s Hospital, The University of Melbourne, Melbourne, Australia; 7 Centre for Allied Health Research and Education, Cabrini Health, Melbourne, Australia; 8 Monash Department of Clinical Epidemiology, Cabrini Institute, Melbourne, Australia; 9 Department of Epidemiology and Preventive Medicine, School of Public Health and Preventive Medicine, Monash University, Melbourne, Australia; 10 Department of Physiotherapy, Curtin University, Perth, Australia; 11 Department of Physical Therapy, Faculty of Rehabilitation Medicine, University of Alberta, Edmonton, Canada; 12 Department of Health Sciences, Macquarie University, Sydney, Australia

**Keywords:** Low back pain, Patient education, Exercise, Self-management, Feasibility, Implementation

## Abstract

**Background:**

Practice-based guidelines recommend patient education and exercise as first-line care for low back pain (LBP); however, these recommendations are not routinely delivered in practice. GLA:D® Back, developed in Denmark to assist clinicians to implement guideline recommendations, offers a structured education and supervised exercise program for people with LBP in addition to a clinical registry to evaluate patient outcomes. In this study we evaluated the feasibility of implementing the GLA:D® Back program in Australia. We considered clinician and patient recruitment and retention, program fidelity, exploring clinicians’ and patients’ experiences with the program, and participant outcome data collection.

**Methods:**

Clinicians (chiropractors and physiotherapists) were recruited and participated in a 2-day GLA:D® Back training course. Patients were eligible to participate if they had persistent or recurrent LBP. Feasibility domains included the ability to: (1) recruit clinicians to undergo training; (2) recruit and retain patients in the program; (3) observe program fidelity; and (4) perceive barriers and facilitators for GLA:D® Back implementation. We also collected data related to: (5) clinician confidence, attitudes, and behaviour; and (6) patient self-reported outcomes related to pain, disability, and performance tests.

**Results:**

Twenty clinicians (8 chiropractors, 12 physiotherapists) participated in the training, with 55% (11/20) offering GLA:D® Back to their patients. Fifty-seven patients were enrolled in the program, with 67% (38/57) attending the final follow-up assessment. Loss to follow up was mainly due to the effects of the COVID-19 pandemic. We observed program fidelity, with clinicians generally delivering the program as intended. Interviews revealed two clinician themes related to: (i) intervention acceptability; and (ii) barriers and facilitators to implementation. Patient interviews revealed themes related to: (i) intervention acceptability; and (ii) program efficacy. At 3 months follow-up, clinicians demonstrated high treatment confidence and biomedical orientation. Patient outcomes trended towards improvement.

**Conclusion:**

GLA:D® Back implementation in Australia appears feasible based on clinician recruitment, program acceptability and potential benefits for patient outcomes from the small sample of participating clinicians and patients. However, COVID-19 impacted patient recruitment, retention, and data collection. To scale-up GLA:D® Back in private and public settings, further work is warranted to address associated barriers, and to leverage facilitators.

**Supplementary Information:**

The online version contains supplementary material available at 10.1186/s12998-022-00427-3.

## Background

Low back pain (LBP) is the leading cause of years lived with disability worldwide [1], imposing an enormous burden on individuals and high societal costs [2]. Over the last 25 to 30 years, the level of disability associated with LBP has risen by more than 50% [2]. Most international guidelines for the management of persistent (equal to or greater than 3 months) or recurrent (repeat episodes) LBP recommend that patients self-manage their condition. First-line patient recommendations include reassurance and education about the nature of their condition, staying at work (or returning as soon as possible), and advising to remain physically active, and/or exercise [3, 4].

Guideline recommendations are not routinely implemented, leading to treatment variability, wasted resources and potential harm [5]. A recent review of evidence-based guideline adherence for musculoskeletal conditions found up to 81% of physical therapists provided care of unknown value, i.e., treatments not mentioned in guidelines [6]. Further, 43% of physical therapists provided low-value care, i.e., treatments that guidelines do not endorse or recommend against [6]. This non-adherence appears to be increasing [7]. Apart from physical therapists, little is known with respect to other health care providers, i.e., chiropractic care aligning with guideline recommendations [8].

To improve the implementation of guidelines into practice for the management of persistent and recurrent LBP, GLA:D® Back (Good Life with osteoArthritis in Denmark) was developed. GLA:D® Back aims to translate guideline recommendations into a practitioner-delivered program to promote evidence-based self-management support [9]. The GLA:D® Back program is presently offered and taught to the members of the chiropractic and physiotherapy professions; and comprises two patient education sessions, plus 16 structured and individualised supervised group exercise therapy sessions [9]. The GLA:D® Back program is based on the successful implementation of clinical practice guideline recommendations for the hip and knee—GLA:D®, with good results for reduced pain, improved function, reduced analgesic intake and reduced sick leave for people with osteoarthritis [10]. GLA:D® Back was developed to adopt a similar approach for those with persistent or recurrent LBP to empower and benefit patients through effective self-management strategies based on their personal goals and capacities [9]. GLA:D® Back has been implemented and evaluated in Denmark [11–15], and more recently, in the Canadian healthcare settings [16]. While the Danish and Canadian programs were deemed practical in their respective settings, using similar feasibility items, both faced similar challenges with adoption, e.g., logistical factors such as setting up the program and patient recruitment. To date, it is not known whether it is feasible to implement this program for LBP in Australia. Despite likely cultural, professional, and legislative differences existing, we hypothesise our results, using similar feasibility criteria, would be comparable to those described in the Danish and Canadian pilot programs [16, 17].

The primary aim of this project was to investigate the feasibility and acceptability of implementing the GLA:D® Back program in Australia. We aimed to evaluate: (1) the ability to recruit chiropractors and physiotherapists to undergo GLA:D® Back training; (2) the ability to recruit and retain patients into the GLA:D® Back program; (3) the ability to directly observe program fidelity; and (4) barriers and facilitators for GLA:D® Back implementation for clinicians and patients. We also collected secondary data pertaining to: (5) clinician confidence, attitudes, and behaviour; and (6) patient self-reported outcomes related to pain, disability, and clinical performance tests.

## Methods

### Ethics

This study was approved by Macquarie University Ethics Board (Project ID 5655): 52019565510186.

### Study participants—clinicians

Chiropractors and physiotherapists were invited to participate from a convenience sample, formed from our clinical networks, assisted by the President of Sports Chiropractic Australia and one of the GLA:D® hip and knee Australia program leads, who both identified clinicians and clinics who had shown interest in, or had enquired about, GLA:D® Back. These clinicians were approached and invited via email and/or phone call by the GLA:D® Back Australia research team to participate in this feasibility study.

Australian Health Practitioner Regulation Agency registered clinicians were eligible to participate if they provided treatment in a primary care setting for people with persistent or recurrent LBP. Clinicians were also required to have access to adequate floor space to conduct patient education groups and group exercise classes, and access to a computer connected to the Internet to enter data into the Research electronic data capture (REDCap) database system [18, 19].

### GLA:D® Back implementation strategy: clinician training

The Australian GLA:D® Back training course was delivered by the developers of the original program in Denmark [9] and local investigators, including the GLA:D® Knee and Hip Australia program lead and prominent Australian LBP primary care researchers. The training ran over 2 days and comprised lectures and practical workshops to educate and motivate clinicians to deliver the program for people with persistent or recurrent LBP [11].

Clinicians were initially exposed to contemporary evidence regarding the burden of LBP to develop or enhance their ability to deliver patient education. Key messages relating to central education themes from the patient education material were introduced (Additional file 1: Appendix Table S1) [9], with clinicians role-playing the delivery of these key messages as part of the education content. Clinicians were taught to facilitate individualised goals, using the Specific, Measurable, Attainable, Realistic and Timed (S.M.A.R.T) approach [20]. GLA:D® Back supervised exercises were also introduced during the training workshop, which blends strength, endurance, and flexibility approaches, divided into four levels of difficulty, and is accompanied by an exercise booklet.

Clinicians were also introduced and given access to a project specific REDCap registry to assist with data collection for patient characteristics and outcomes. The platform also contained essential GLA:D® Back materials, comprising a slide show to be used for patient education, exercises to support patients’ reflections, and posters with key patient education messages. Implementation was further supported through a private group messaging App for ongoing, informal clinician feedback. Clinicians had access to the research team to express concerns or ask questions. Additionally, an online 1-h booster session was conducted 3 months after initial training to refresh the clinicians’ knowledge of the GLA:D® Back course material and to encourage patient recruitment.

### Study participants—patients

Adults (18 years or older) reporting persistent or recurrent LBP were eligible to participate in the GLA:D® Back program. Patients currently under care at the clinic could participate in GLA:D® Back and if deemed relevant, continue other therapy, or care concurrently. The study was limited to participants with adequate English comprehension to complete the study questionnaires. Exclusion criteria included: first episode of acute LBP (less than 3 months duration); nerve root involvement/clear neurologic signs; suspected serious pathology; spinal trauma (e.g., fracture); pregnancy; conditions that require a specialist consultation or could be aggravated or worsened if treated by the interventions in this study. Patients with planned spinal surgery or other major surgery within the study period were excluded, as were patients currently participating in a supervised exercise or rehabilitation program.

Clinicians were directed to approach, screen, and recruit patients who they considered would benefit from improved self-management skills for recurrent or persistent LBP. Interested patients provided their email addresses and received study information and an online form in REDcap by which to provide consent. Patients who were interested in participating were required to sign informed consent at the clinic prior to commencing the program. Recruited patients completed outcome measures for the clinical registry at baseline, 8 weeks and 3 months follow up.

### GLA:D® Back intervention

Eligible patients performed four clinical performance tests, supervised by their clinician: the standing forward bending test [21], trunk flexor endurance test [22, 23], back extensor endurance test [22–24], and 30 s chair stand test (Additional file 2: Appendix Table S2) [25]. S.M.A.R.T. goals were also discussed and established as was the exercise program with the initial level of difficulty determined. As the GLA:D® Back intervention was developed around cognitive behavioural theory [9], patients commenced the program with education classes in the clinic over 2 1-h sessions. Key education themes were introduced to help patients understand recurrent and persistent LBP in a non-threatening way. This was followed by the supervised exercise program, which comprised 16, 1-h, individualised exercise sessions in a patient group setting bi-weekly over an 8-week period. Clinicians guided and encouraged patients to focus on: (i) exploring variation of movements; (ii) feeling key muscles during the exercises; and (iii) motivation and confidence building [9]. Clinicians conducted a final patient assessment and interview, revisiting personalised goals and clinical performance tests. Further details of the GLA:D® Back training and intervention has been described and published previously [9].

### Feasibility outcomes

Assessing feasibility of implementation were based on the following 4 domains.

#### Addressing aim 1: clinician recruitment

We aimed to recruit 10-to-20 interested clinicians for the GLA:D® Back course within 2 weeks of contacting them. Of those recruited, we defined feasibility success if 50% of clinicians delivered the program.

#### Addressing aim 2: patient recruitment, retention, and data collection

Clinicians aimed to recruit as many eligible patients as possible within a 3-month period. Additionally, we planned to record patient retention rates over the course of the program offered within each clinic and assess the feasibility of collecting patient outcomes at baseline, 8 weeks, and 3 months from the GLA:D® Back program commencement. Recruiting a minimum of 60 patients, with ≥ 80% retention rate (i.e., completing the program) would meet our criterion for feasibility success.

#### Addressing aim 3: observation of delivery of the program

We aimed to observe and document elements of fidelity, i.e., adherence related to delivery of the program, including patient attendance, absences, and cancellations, as well as observing session length, evidence of individualised coaching and group discussions. We planned to observe and document the presence of materials used, such as visibility of the patient training manual, GLA:D® Back key messages poster, PowerPoint slides and clinician use of reflection exercises. Items of the fidelity checklist can be found at Additional file 3: Appendix Table S3.

#### Addressing aim 4: barriers and facilitators

We aimed to explore barriers and facilitators for GLA:D® Back implementation through qualitative interviews with both clinician and patient participants. Interview questions were informed by the Theoretical Domains Framework (TDF) [26], to identify factors influencing healthcare provider behaviour. Three researchers (MF, AY, JW) conducted one-to-one semi structured interviews online (Zoom) or via telephone, which were audiotaped and transcribed.

One clinician from each participating clinic who delivered GLA:D® Back will interviewed with questions focussed on perspectives regarding the content of the clinical intervention and program implementation and feasibility. Patients who both completed and dropped out of the program were interviewed and asked to reflect on their experiences and what they gained by participating in the GLA:D® Back program. In developing the patient interview questions, a consumer who had experience with LBP from Musculoskeletal Australia (www.msk.org.au) was invited to comment on the questions related to the patient’s LBP experience.

#### Secondary aims 5 and 6: clinician and patient self-reported outcomes

Clinician outcome measures were collected 1 week before, immediately after, and 3 months post-course to evaluate the potential change in the Practitioner Confidence Scale [27], along with biomedical and behavioural treatment orientation within the practitioner attitudes and beliefs scale [28, 29]. Patients received a link to the REDCap system on the day of the baseline consultation and 3 months later, which included the following outcomes: Arthritis Self-Efficacy Scale [30], Oswestry Disability Index = ODI [31], Numeric Pain Rating Scale [32], the Fear-Avoidance Behaviour Questionnaire—FABQ [33], the STarT Back Screening Tool [34], the Brief Illness Perception Questionnaire—B-IPQ [35], the SF-36 quality of life questionnaire [36] and self-assessed physical fitness [37]. Details of all outcome measures and interpretation scales are shown in Additional file 2: Appendix Table S2.

### Analysis

Quantitative data were presented descriptively, including: (1) clinician recruitment rates; (2) patient recruitment, retention and follow up rates; and (3) directly observing the GLA:D® Back program. Qualitative data analysis occurred via codes generated and grouped into themes using thematic analysis from the qualitative semi-structured interviews [38].

Researchers (MF, AY and JW) conducted a qualitative analysis of each interview transcript by individually reading line by line to capture an overall impression of the data. Data codes, supported by Nvivo software (QSR International Pty Ltd, Melbourne, Australia), were collated into a broader level of potential themes. Subsequent ensuing themes were discussed until a consensus was reached by researchers (MF, AY, JW). Themes related to barriers and facilitators were then mapped to the relevant domains from the TDF [26]. Direct quotations from the main themes are presented.

For clinician outcomes, group median change scores were calculated on the Practitioner Confidence, and Practitioner Attitudes and Beliefs Scales at baseline, post training and 3- month follow-up. Patient outcomes in terms of pain, disability, fear avoidance behaviour, prognosis, brief illness perception, quality of life and physical fitness were also described as median change scores from baseline to 3 months. As this was a feasibility study, no inferential analyses were performed.

## Results

### Clinicians and patient baseline demographics

Eight chiropractors and 12 physiotherapists from 11 individual clinics participated in the 2-day workshop at Macquarie University, Sydney, Australia in November 2019. Clinicians were 35% female, with average age 36 years. Seventy percent of clinicians who delivered the GLA:D® Back intervention were very satisfied with the program. Clinician characteristics are described in Table 1. Briefly, clinicians favoured a biomedical treatment orientation at baseline (Table 2).

**Table 1:** Clinician characteristics and outcomes (N = 20)

	N (%) (unless other specified)
Age, mean (range)	36 (23–52)
Female	7 (35%)
Physiotherapist	12 (60%)
Chiropractor	8 (40%)
*All clinicians*	
Clinic owner	5 (25%)
Self-employed	6 (30%)
Employee	6 (30%)
Other (i.e., contractor)	3 (15%)
Private clinic	16 (80%)
Hospital department	4 (20%)
*Clinical experience*	
0–5 years	6 (30%)
6–10 years	2 (10%)
11–20 years	7 (35%)
> 20 years	5 (25%)
*Previous experience with GLA:D® hip and knee*	
No experience	9 (45%)
Have referred to GLA:D in house (within the clinic)	1 (5%)
Have referred to GLA:D in another clinic	0 (0%)
Have instructed GLA:D groups	9 (45%)
Other	1 (5%)
*Training evaluation, immediately after the GLA:D® Back course—median (range)*	
Content (0–10) range	9 (8–10)
Usability (0–10) range	9 (8.5–10)
Novelty (0–10) range	8 (8–9)
*Overall satisfaction with the GLA:D® Back program*	10 missing
Very satisfied	7 (70%)
Satisfied	3 (30%)
Neither satisfied nor dissatisfied	0 (0%)
Dissatisfied	0 (0%)
Very dissatisfied	0 (0%)
*Satisfaction with patient education materials*	10 missing
Very satisfied	4 (40%)
Satisfied	5 (50%)
Neither satisfied nor dissatisfied	0 (0%)
Dissatisfied	1 (10%)
Very dissatisfied	0 (0%)

**Table 2:** Clinician outcomes

	Baseline—pre training (n = 20)	Follow-up post-training (n = 19)	Adjusted for clinicians who delivered the program (n = 11)	Difference in median: post–pre	Difference in median: post–pre (Adjusted for clinicians who delivered the program n = 11)
	Median	Median	Median	Median	Median
PCS	14	13	14	− 1	0
PABS (biomedical)	46.5	46	49	− 0.5	3
PABS (behavioural)	27.3	27	27	− 0.3	− 0.3

PCS: Practitioner Confidence Scale (ranges from 0–40); higher scores indicating lower confidence

PABS: Practitioner Attitudes and Beliefs Scale (ranges from 10–60 for biomedical, 9–54 for behavioural); higher scores indicate a more biomedical or behavioural orientation, respectively

Patients were 67% female, with an average age 57 years. Fifty-nine percent of patients reported experiencing LBP for more than 1 year, with 29% taking over the counter medication, and 14% prescription medication (Table 3). Fifty-one percent of patients were deemed high risk for a poorer prognosis according to the the STarT Back Screening Tool (Table 3), while fear avoidance behaviour was elevated at baseline with a median value of 15 (range 0–24) [39] (Table 4).

**Table 3:** Patient characteristics (N = 57)

Total patients	Baseline N (%) (unless other specified)
Female	34 (66.7%)
Age, mean (SD) years	56.6 (14.1%)
Height, mean (SD) cm	167 (17.5%)
Weight, mean (SD) kg	85 (22.5%)
*Level of education*	
No qualification	1 (2%)
Vocational training	11 (22%)
Higher school education (year 10)	7 (14%)
Higher school education (year 12)	4 (8%)
Medium higher education (e.g., Bachelors)	22 (44%)
Long higher education (e.g., Masters or PhD)	4 (8%)
*Employment status*	
Full-time	16 (33.3%)
Part-time	8 (16.7%)
Casual	4 (8.3%)
Unemployed	1 (2.1%)
Retired	16 (33.3%)
Housewife/other	3 (6.3%)
*Clinical symptoms/pain duration*	
0–2 weeks	3 (5.9%)
2–4 weeks	0 (0%)
4–12 weeks	6 (11.8%)
3–12 months	12 (23.5%)
> 1 year	30 (58.8%)
*Previous episodes (n* = *51)*	
0	10 (19.6%)
1	6 (11.8%)
2–3	11 (21.6%)
> 3	24 (47.1%)
Missing	6
*Time since treatment initiated (n* = *51)*	
< 2 weeks	5 (9.8%)
2–4 weeks	4 (7.8%)
> 4 weeks	43 (82.4%)
Missing	6
*No. of healthcare visits for low back pain (n* = *51)*	
1	20 (39.2%)
2–5	23 (45.1%)
6–10	7 (13.7%)
> 10	1 (2%)
Missing	6
*Pain medication (n* = *51)*	
None	29 (56.9%)
Over the counter	15 (29.4%)
Prescription	7 (13.7%)
Missing	6
*STarT Back risk (n* = *50)*	
Low	5 (10%)
Medium	19 (39%)
High	25 (51%)
Missing	7

**Table 4:** Patient outcome measures

Measure	Baseline Median	3-months Median	Pre-post median change	Participants at Baseline	Participants at follow up
Brief illness perceptions (B-IPQ 0–80)	48	46.5	1.5	n = 50, 7 missing (14% missing)	n = 40, 17 missing (43% missing)
Fear-avoidance beliefs (FABQ 0–24)	15	7	7	n = 53, 4 missing (8% missing)	n = 38, 19 missing (50% missing)
Perceived physical fitness (0–40)	18.5	20	1.5	n = 52, 5 missing (10% missing)	n = 40, 17 missing (43% missing)
Seconds of trunk flexor endurance (0–120)	43.5	102	58.5	n = 54, 3 missing (6% missing)	n = 38, 19 missing (50% missing)
Seconds of extensor endurance (0–180)	67	159	92	n = 56, 1 missing (2% missing)	n = 38, 19 missing (50% missing)
Sit to stand test—30 s	10	13	3	n = 55, 2 missing (4% missing)	n = 37, 20 missing (54% missing)
Oswestry disability index (ODI 0–100)	44.4	36	− 8.4	n = 53, 4 missing (8% missing)	n = 40, 17 missing (43% missing)
Back pain (0–10)	5	3	− 1.5	n = 51, 6 missing (12% missing)	n = 41, 16 missing (39% missing)
Leg pain (0–10)	2	1	− 1	n = 51, 6 missing (12% missing)	n = 39, 18 missing (46% missing)

B-IPQ (0–80); higher scores indicate more threatening views

FABQ (0–24); higher scores indicate more fear-avoidance beliefs

Perceived physical fitness (0–40); higher scores indicate better perceived fitness

Seconds of trunk flexor endurance (0–120 s); holding the position for as long as possible up to a maximum of 2 min

Seconds of extensor endurance (0–180 s); holding the position for as long as possible up to a maximum of 3 min

Sit to stand test (0–30 s); as many repetitions as possible in 30 s

ODI (0–100); higher scores indicate more disability

Back pain (0–10); lower scores indicate lower levels of pain

Leg pain (0–10); lower scores indicate lower levels of pain

### Feasibility outcomes

#### Clinician recruitment (aim 1)

Thirteen clinicians were initially approached, with 9 recruited within 2 weeks of being contacted to undergo GLA:D® Back training. Twenty clinicians from 12 clinics were recruited within 4 weeks.

Eleven of the 20 clinicians (55%) adopted the GLA:D® Back program, meeting our feasibility criteria (50%) with 8 of 12 (67%) clinics recruiting patients. Of the 9 clinicians who were unable to implement the program, two clinicians from one clinic had difficulty obtaining further ethics approval specific to their workplace (hospital setting), two clinicians from one clinic recruited patients but did not commence due to COVID-19, and two clinics, one physiotherapy (1 clinician) and one chiropractic (2 clinicians) had recently relocated to new premises and no longer had access to the patients they intended to recruit. Finally, in one chiropractic clinic, two of the three participating clinicians did not recruit patients. No reason was provided.

#### Patient recruitment, retention, and data collection (aim 2)

Apart from one clinic that commenced recruitment immediately after the GLA:D® Back training, patient recruitment for most clinicians began within 3 months following program delivery. This was based on the upcoming holidays, considered likely to impact patient recruitment and retention over this period.

A total of 57 patients were recruited by the 11 clinicians who implemented the GLA:D® Back program, just short of our feasibility criterion of 60 patients. Between 88 to 98% of patients provided baseline data from questionnaires, depending on the outcome measure. Of the participants recruited, 67% (38/57) attended the last treatment session and final assessment follow-up 8 weeks later. Similarly, 67% (38/57) completed the 3-month follow-up survey. Ninety-two percent of patients (35/38) participated in more than 80% of the GLA:D® Back program sessions. Of the enrolled participants, 33% (19/57) dropped out for various reasons, with the impact of COVID-19 being the most dominant reason. We therefore did not meet our feasibility criterion of ≥ 80% retention.

#### Observation (aim 3)

We directly observed one exercise group session in person and three sessions online (due to COVID-19) at four different sites (a total of four clinicians and 15 patients). From our fidelity checklist (Additional file 3: Appendix Table S3), in two classes, the duration of the sessions did not reach 60 min (45 and 50 min respectively) and the number of exercises completed by patients varied, i.e., they were not all performed. All clinicians delivered individualised coaching and generated group discussions. In relation to GLA:D® Back materials used, the key messages poster was notably absent in the four exercise classes observed. All but one patient training manual was observed.

#### Barriers and facilitators (aim 4)

##### Clinician interviews

Barriers and facilitators to the GLA:D® Back program were related to several domains of the TDF. Interviews averaged 30 min in duration.

##### Theme (1): acceptability of the intervention to clinicians.

GLA:D® Back training was highly acceptable to these physiotherapists and chiropractors. All clinicians described a positive experience with the 2-day training program. The components of the program (education and exercise) were described as evidence-based and a structured package that would facilitate optimal care for their patients.

Overall, it was a very positive experience. I enjoyed learning something that was bringing clinical practice guidelines into practice. (Physiotherapist)

It’s an evidence-based program that works, I think in a nutshell. (Chiropractor)

##### Theme (2): barriers and facilitators

Several clinicians reported finding it easy to implement GLA:D® Back. They described the following facilitators: having a referral network (GPs, other allied health practitioners); having demand (e.g., clinics who already offered GLA:D® hip and knee or another form of group exercise); having a short list of patients to invite to participate; keeping the cost low to patients; having the resources ready to go, including an online platform to deliver the program during the COVID-19 pandemic.

The training prepared us well to deliver it. The minimal equipment is great, and … when COVID occurred was being able to do it via Zoom. (Physiotherapist)

A strong referral base where doctors trust what you’re doing…. What made it easy was the really good information…. That we could send out to doctors when we were looking for people to be part of the pilot program. (Physiotherapist)

For the clinicians who had difficulties implementing GLA:D® Back, a common barrier was difficulty with patient recruitment. Clinicians described being slow to start or recruit, not having a rolling recruitment, and not having an adequate marketing strategy. A lack of administrative support made it challenging to start the program, including the time required to prepare the equipment needed, patient logbooks and educational resources. Other barriers included: scheduling challenges (out of clinic hours/peak clinic hours), the upfront cost of the program to patients, hospital ethics and the COVID-19 pandemic.

We had one that had lost his job, so couldn’t afford it… a couple that probably weren’t interested in in doing it virtually (Physiotherapist)

I think the biggest thing comes down to the way we marketed it, and probably not having the time to explore different forms of marketing to get a better response (Chiropractor)

#### Patient interviews

Two dominant themes emerged from the patient interviews that related to feasibility of implementing GLA:D® Back: (1) acceptability of GLA:D® Back as an intervention; and (2) effectiveness of the program.

##### Theme (1): acceptability of GLA:D® Back as an intervention

Most of the patients thought GLA:D® Back was practical and great, describing the program using descriptors such as ‘fantastic’, ‘simple’ and ‘manageable’ ‘essential and ‘enlightening’.

It’s absolutely fantastic…. You don’t need any special equipment… it’s something you can take you know in your normal life.

Getting that concept across that pain has got nothing to do with damage and it was really enlightening actually. And also, there was a bit of emphasis on you need to be able to do all movements you can’t be stopping movements. Which was true for me where you’ve been told not to do years ago.

Most of the patients spoke positively about the group aspect fostering some healthy competition/encouragement and said that being supervised by a clinician made them feel safe, exploring new movements.

It was good great to do it as a group…to motivate me to do it… you can ask a question and if you’re not sure about it I think it helps other people.

Patients compared their experience of face-to-face to the online mode of delivery, and the transition to online worked well for most patient, but not for everyone.

Definitely a few classes in front of them (clinicians) and get some confidence and then go back to virtual.

People are starting to feel comfortable, but I think the timing of the transition ruined that… we just haven’t gone on long enough to sort of recapture it via zoom.

##### Theme (2): perceived benefits of the program

Most patients reported a variety of benefits such as an increase in confidence in their ability to manage their back, increased strength, a reduction in pain (except for soreness after some of the sessions), and a reduction in the number of flare-ups.

It’s definitely given me the confidence to try new things. And do things and push myself further then I would have before.

I feel much more confident doing things. That if I do get a little flare-up it’s not going to hold me back like it did before.

##### Secondary clinician and patient outcomes (aim 5 and 6)

Clinicians were very satisfied with the GLA:D® Back program (70%) and satisfied/very satisfied with the patient education materials (90%) (Table 1). Practitioner confidence remained unchanged from baseline, while the practitioner attitudes and beliefs scale showed a 3-point change towards biomedical treatment orientation at 3 months follow up (Table 2). Behavioural orientation remained unchanged.

From baseline measures, patients reported improved median change of 7 points on the FABQ, 8.4 points on the ODI, 1.5 points for back pain and 1 point for leg pain (Table 4). Objectively measured clinical performance tests demonstrating a median improvement of 59 s on the trunk flexor test, 92 s on the extensor endurance test and 3 repetitions on the 30 s sit to stand test (Table 4).

## Discussion

Our findings suggest that the implementation of GLA:D® Back appears feasible in Australian physiotherapy and/or chiropractic private practice settings. The program was acceptable to the small sample of invited clinicians with potential improvement in outcomes by the small sample of participating patients. However, the COVID-19 pandemic impacted patient recruitment, follow-up rates and data collection. Strategies to address these need to be put into practice to scale up GLA:D® Back implementation.

We recruited 20 enthusiastic and motivated clinicians, including physiotherapists who had previously implemented GLA:D® hip and knee. However, only 11 of the 20 clinicians (55%) adopted the GLA:D® Back program. This met our pre-determined success criteria (50%), and is in line with the GLA:D® Back 2019 Danish Annual Report of clinicians conducting the program (54%) [40]. However it was lower than the Canadian GLA:D® Back program, with 71% of their clinicians delivering the program [16]. While clinicians from both the Australian, Danish and Canadian programs experienced similar barriers implementing the program, it is highly likely the COVID-19 pandemic further impacted some of our clinician’s capacity to offer GLA:D® Back. Importantly, clinicians who did not deliver GLA:D® Back in Australia expressed the intention to deliver the program in the future if given the opportunity.

We successfully recruited 57 patients, just short of our minimum criterion of 60. However, retention was impacted by a 33% dropout rate, thus not meeting our predefined criterion of ≤ 20% of dropouts. This contrasted with the Canadian program, which reported a 12% drop out rate [16]. The COVID-19 outbreak was largely responsible for dropouts in our study, accounting for almost 50% of our patient dropouts. Other contributing factors were barriers to patient recruitment, i.e., programs were suspended or paused for patient safety or abandoned due to business restrictions imposed by the Federal Government. A transition to online delivery (via Zoom) was offered and generally well accepted by most patients. Amendments to the original face-to-face program were few, with clinical performance tests observed online, and patient education continuously reiterated, with clinicians providing patient feedback virtually. Some patients declined virtual delivery finding the transition either too difficult or were not interested in this format.

Of our 57 participants, 35 of 38 (92%) who remained in the program took part in more than 80% of the GLA:D® Back program sessions available, suggesting good program adherence. This is comparable to the Canadian program, where 84% of their participants attended both education sessions and 74% attended the majority (if not all) 16-session within the program [16].

The patient attrition rate impacted completeness of patient-reported items, with 67% of patients completing the 3-month follow-up survey. This rate was identical to the Canadian program [16], but short of the 88% response rate for GLA:D® Back participants in Denmark [17]. As a pragmatic implementation study, a response to every question was not mandatory, hence, we do not have the entire patient population responding to some questions, including the SF-36 and sick leave follow-up data. Future studies will need to investigate if non-response introduces bias, while concurrently seeking ways to improve follow-up rates and responses to clinical outcomes. This could include altering the patient reminder system (adding phone text reminder messages and phone calls to standardised emails) and/or building better rapport (i.e., checking up) with participants.

We did not observe the key education messages poster during all four clinic fidelity observations, despite one or more key messages from these posters delivered during the classes by the clinician. The GLA:D® Back key messages poster is recommended for the education sessions and integrated into the exercise sessions [9]. Posters may have been visible in another session not observed or were not visible from the Zoom screen sharing. Further, some exercises were either not performed or were modified by clinicians, who accommodated patients by adapting to their injury history and/or observed comorbidity. Future GLA:D® Back offerings may consider further regression of exercises within the level of difficulty currently offered.

### Facilitators and barriers of the GLA:D® Back program

Most clinicians were confident, motivated, and freely volunteered for the course. GLA:D® Back implementation was smooth, particularly for those who previously implemented systems associated with the GLA:D® hip and knee program, i.e., had dedicated floor space and established referral networks. Clinicians observed several patient benefits, including clinical performance test improvements at follow-up, new knowledge gained and learning to move ‘more freely’. Patients felt more confident handling their back problems despite the ongoing presence of pain and future flare ups, underscoring one of the desired effects of the GLA:D® Back program. These apparent patient beliefs observed quantitatively via the FABQ and behavioural changes observed by clinicians should be further investigated for maintenance over the longer-term. Patients also felt reassured, with clinicians nurturing a safe environment, promoting a friendly, competitive atmosphere in a group setting [41]. As such, group exercises should be further encouraged, in light of group-based and individual physiotherapy exercise programmes being found to be equally effective for musculoskeletal conditions [42].

Some clinicians encountered difficulties implementing the GLA:D® program, including patient recruitment challenges. This underscores the importance of skilled staff and other resources dedicated to patient recruitment (i.e., implementing marketing strategies and assisting program set up). Clinicians undertaking such roles were likely discouraged to implement the program, being drawn away from their established clinical responsibilities. Greater support provided by the GLA:D® Back team (beyond the training course) may facilitate greater widespread adoption of GLA:D® Back among clinicians.

Virtual platforms were not adopted by all patient in our GLA:D® Back study, with some favouring the initial face-to-face approach of the program. Emerging evidence [43], indicates that telehealth can provide improvements in exercise adherence [44]; however, patient barriers like age, education level and computer literacy can all hinder virtual uptake [45]. Future research should look to overcome telehealth barriers, in light of a recent survey showing that virtual participation facilitated the clinicians’ role as coaches [46], consistent with the GLA:D® back self-management approach.

### (Secondary) clinical and patient outcomes

Clinicians’ overall evaluation of the course was positive, reflected by successful adoption of the program. Clinician treatment orientation (beliefs and attitudes) favoured a more biomedical orientation at 3 months follow up, contrasting the GLA:D® Back studies in Denmark [17], Canada [16], and the overall GLA:D® Back objectives. It may be that our clinicians held initial strong biomedical beliefs [47, 48], reflective of their training [49], or being perceived as not meeting patient treatment expectations [50]. Almost 60% of patients reported experiencing LBP for more than 1 year, therefore they were appropriately recruited for the GLA:D® Back program. Improvements in fear avoidance suggests the program’s education and exercise modules may be addressing this component. Positive changes were noted for the clinical performance tests, which are in line with patient improvements in the Canadian [16] and Danish programs [40]. Patient illness beliefs (B-IPQ) did not meaningfully change over time and may likely altered over a longer time duration or not at all. While results from our outcomes are underpowered, they show promise, with trends like those reported in previous GLA:D® Back programs [16, 17, 40].

### Study strengths and limitations

A key limitation of our study implementation was the impact of the COVID-19 pandemic. As a feasibility study, only preliminary short-term evaluation of clinician and patient outcomes took place, hence the maintenance of long-term outcomes is unknown. We also conveniently sampled and recruited clinicians we felt would be interested in and likely adopt the program. Although evidence and guidelines support the approach and intervention taken with GLA:D® Back, no clinical trial has evaluated efficacy of the program compared to usual care. The small sample in our feasibility study signifies a need to evaluate implementation with a larger sample of clinicians and patients prior to a national roll out of the program. Implementation of our study was limited by the in-person training of clinicians, i.e., limited by cost and location, and there is a need to train local trainers to deliver the program. The feasibility of online training was also not assessed. Further, inclusion of other stakeholders in qualitative research is needed, including potential referrers (e.g., general practitioners), and funders (e.g., private health insurers). We only assessed some aspects of fidelity, with future fidelity assessments targeting focused behavioural change techniques and clinician communication styles. While our secondary aims were pertaining to patient outcomes, it should be noted that improvement trends in these outcomes cannot be taken as evidence for effectiveness. Our feasibility study in Australia builds on the original English implementation of the GLA:D® Back program in Canada [16], therefore encouraging implementation in other English-speaking countries.

## Conclusion

Implementation of the GLA:D® Back program in Australia appears feasible. The program was acceptable to the small sample of invited chiropractors and physiotherapists and with potential improvement in patient outcomes. However, the COVID-19 pandemic impacted patient recruitment, retention, and data collection. To scale up GLA:D® Back implementation in private and public settings, further work is warranted to address associated barriers, and to leverage facilitators. Also, clinical benefits of the program when compared to current models of care needs to be determined.

## Supplementary Information

**Additional file 1. Appendix Table S1**. Key messages in GLA:D® Back used during patient education and repeated during the exercise sessions to encourage self-management.

**Additional file 2. Appendix Table S2**. Overview of clinician and patient outcome measures.

**Additional file 3. Appendix Table S3**. Fidelity elements of the clinical intervention to be observed.

## Data Availability

The datasets used and/or analysed during the current study are available from the corresponding author on reasonable request.
