# Supplementary material for: GLA:D® Back Australia: a mixed methods feasibility study for implementation
Source: Chiropr Man Therap. 2022 Apr 7;30:17. doi: 10.1186/s12998-022-00427-3 (PMC8989099; doi:10.1186/s12998-022-00427-3)
Supplement: Supplementary file 1 — Additional file 1. Appendix Table S1. Key messages in GLA:D® Back used during patient education and repeated during the exercise sessions to encourage self-management. [file 12998_2022_427_MOESM1_ESM.docx]

**Appendix Table S1**

**Eight themes or key messages from the patient education material:**

1) Posture and spinal abnormalities are common

2) Pain equals alarm - not harm

3) The spine is made for movement

4) Natural movements inhibit pain

5) Training strengthens the back

6) Action precedes improvement

7) The back is strong

8) The brain can turn the pain up or down
