# Supplementary material for: GLA:D® Back Australia: a mixed methods feasibility study for implementation
Source: Chiropr Man Therap. 2022 Apr 7;30:17. doi: 10.1186/s12998-022-00427-3 (PMC8989099; doi:10.1186/s12998-022-00427-3)
Supplement: Supplementary file 2 — Additional file 2. Appendix Table S2. Overview of clinician and patient outcome measures. [file 12998_2022_427_MOESM2_ESM.docx]

**Appendix Table S2** Clinician and patient outcomes

| **Clinician outcomes: course evaluation, confidence, attitudes, and behaviour** | | |
| --- | --- | --- |
| **DOMAIN** | **OUTCOME** | **DESCRIPTION** |
| GLA:D Back training 2-day course evaluation | 3 domains: (i) course content, (ii) degree of novelty and (iii) usability | (0 = very poor and 10 = excellent), as well as satisfaction with the training course and program (0 = very unsatisfied; 10 = very satisfied). In addition, satisfaction with education materials were also scored on a scale of 0 to 10 (0=very unsatisfied and 10=very satisfied). |
| Clinician’s confidence | Practitioner Confidence Scale or PCS | 4-item scale to score practitioner confidence, with each item scored on a 5-point scale (1 = strongly agree to 5 = strongly disagree), with higher scores indicating lower confidence |
| Clinician attitudes and beliefs | Practitioner Attitudes and Beliefs Scale (PABS) | Ten items comprise the biomedical subscale (sum score 10–60) and the behavioural subscale comprise of 9 items (sum score 9–54), and each were scored on a 6-point scale (1 = totally disagree to 6 = totally agree). Higher scores indicate a more biomedical or behavioural orientation, respectively |
| **Patient outcomes** | | |
| Clinical tests - Physical back performance | 1. Standing Forward Bending Test | 4 = no pain with normal movement, 3 = pain and normal movement, 2 = no pain with abnormal movement, 1 = pain and abnormal movement, 0 = test not completed |
|  | 2. trunk flexor endurance test | 0 seconds holding the position for as long as possible up to a maximum of 2 minutes in static sit-up position. |
|  | 3. extensor endurance test | static extension from 0 seconds holding the position for as long as possible up to a maximum of 3 minutes. |
|  | 4. sit to stand test | number of repetitions of standing from seated in 30 seconds |
| Self-efficacy | Arthritis Self-Efficacy Scale (ASES) | ‘Pain’ (ﬁve questions), ‘Function’ (nine questions) and ‘Other’ (six questions). Each range from 0-10, 0 = very uncertain, 10 = very certain |
| Activity limitation / disability | Oswestry Disability Index | 0–100; higher scores reflect more disability |
| Pain intensity | Numeric Pain Rating Scale | For LBP and leg pain, 0 = no pain, 10 = worst imaginable pain |
| Fear of movement | Fear-Avoidance Behaviour Questionnaire | 0–24; higher scores reflect more fear-avoidance beliefs |
| Risk profile | STarT Back Screening Tool | low risk, medium risk, high risk of poor prognosis |
| Illness perceptions | Brief Illness Perception Questionnaire | range 0–80; higher scores reflect more threatening view |
| Quality of life | SF-36 quality of life questionnaire | 0–100; 0 = very poor, 100 = very good |
| Perceived physical fitness | Physical fitness | Sum score of self-assessed strength, endurance, cardiovascular fitness, balance, range 0–40; higher scores reflect better perceived fitness |
| Pain medication | Pain medication | Current use of over the counter or prescribed pain medication documented as a yes/no binary response |
