# Supplementary material for: GLA:D® Back Australia: a mixed methods feasibility study for implementation
Source: Chiropr Man Therap. 2022 Apr 7;30:17. doi: 10.1186/s12998-022-00427-3 (PMC8989099; doi:10.1186/s12998-022-00427-3)
Supplement: Supplementary file 3 — Additional file 3. Appendix Table S3. Fidelity elements of the clinical intervention to be observed. [file 12998_2022_427_MOESM3_ESM.docx]

**Appendix Table S3** fidelity checklist

| **Fidelity checklist** | Clinician A | Clinician B | Clinician C | Clinician D |
| --- | --- | --- | --- | --- |
| **Structural observations** |  |  |  |  |
| Session number |  |  |  |  |
| Number of patients attending the class |  |  |  |  |
| Number of cancelations |  |  |  |  |
| Number of absences |  |  |  |  |
| Session length (minutes) |  |  |  |  |
| Participants all received individual coaching |  |  |  |  |
| Group discussions facilitated |  |  |  |  |
| **GLAD back material used** |  |  |  |  |
| Clinician refers to key message’s poster |  |  |  |  |
| Clinicians used PowerPoint slides |  |  |  |  |
| Clinicians use worksheets |  |  |  |  |
| Clinician refers to training manual content |  |  |  |  |
| Clinician uses reﬂection exercises |  |  |  |  |
